# Analysis of Recipes Shared as ‘Healthy’ in a Popular Brazilian Website: A Cross-Sectional Study

**DOI:** 10.3390/ijerph192113914

**Published:** 2022-10-26

**Authors:** Anice Milbratz de Camargo, Alyne Michelle Botelho, Gabriella Beatriz Irmão, Giovanna Medeiros Rataichesck Fiates

**Affiliations:** Nutrition in Foodservice Research Centre, Graduate Program in Nutrition, Universidade Federal de Santa Catarina, Campus Universitário João David Ferreira Lima—Trindade, Florianópolis 88040-900, Santa Catarina, Brazil

**Keywords:** internet recipes, food processing, culinary recipes, website, qualitative framework

## Abstract

Cooking is crucial to the achievement of healthy eating habits, and the internet, as host of culinary recipes websites, is a medium for the dissemination of cooking-related content. Research has revealed that most recipes available on internet sites do not have healthy characteristics when compared to recommendations for healthy eating, even the ones promoted as ‘healthy’. This study investigated culinary recipes available on the ‘healthy eating’ section of a popular Brazilian recipe-sharing website. Recipes (*n* = 814) were analyzed with a validated framework based on national dietary guidelines. Ingredients (*n* = 5887) were classified according to the extension and purpose of their industrial processing. The recipes’ titles were content analyzed to identify the health-related words and phrases used. Recipes contained ultra-processed foods and not enough unprocessed or minimally processed foods, such as legumes (4.7%, *n* = 380), nuts and seeds (18.4%, *n* = 150), and fruits (*n* = 32.7%, *n* = 263). The recipes’ titles mentioned 564 health-related terms, appealing to physical characteristics, including weight loss, and fads, such as gluten-free, dukan, low-carb, detox, fitness, ripped body, and belly burner. Therefore, the ‘healthy’ recipes available on the Brazilian recipe-sharing website presented many aspects not in accordance with national dietary guidelines.

## 1. Introduction

Food is one of the basic requirements for the promotion and protection of health, acting as a determining factor in the prevention of chronic non-communicable diseases [1]. The first edition of the Dietary Guidelines for the Brazilian Population brought recommendations for healthy eating based on the classification of food into different groups: cereals, tubers, and roots; fruits and vegetables; legumes; milk and dairy products, meat and eggs; fats, sugars, and salt. It also provided guidelines on the importance of the quality of food ingested in each food group, in addition to orienting the greater consumption of some foods considered protective (fruits and vegetables) and the restriction of others that are rich in trans-fats, sugar, and salt [2]. In the most recent edition, the Guidelines’ recommendations are based on the extension and degree of processing to which foods are subjected before their acquisition, preparation, and consumption. The classification provides four food categories: unprocessed and minimally processed foods; processed culinary ingredients; processed foods; and ultra-processed foods. The consumption of ultra-processed (UP) foods should be avoided, as they consist of formulations of ingredients which are usually nutritionally unbalanced, as well as rich in fats, sugars, and substances of exclusive industrial use, while being poor in fiber and micronutrients [1,3].

Conversely, a healthy diet is one based on unprocessed or minimally processed foods and on the culinary preparations they are made with, seasoned and cooked in moderation with the addition of processed culinary ingredients, such as salt, sugar, and fats [1,3]. There is a body of literature to support this recommendation, showing that cooking more at home with unprocessed or minimally processed foods is related to a better-quality diet [4,5,6,7,8,9]. In fact, the Dietary Guidelines for the Brazilian Population was the first to explicitly promote cooking as a strategy to improve healthy eating, encouraging individuals to appreciate and value it as a social and cultural practice [1]. Nevertheless, for this to happen, some knowledge on cooking is required, which is why the guidelines of Brazil and many other countries’ for healthy eating encourage individuals to learn and share cooking skills with other people [1,10,11,12,13].

Specifically, the Dietary Guidelines for the Brazilian Population recommend looking up recipes on the internet as one way to develop cooking skills [1]. Culinary recipes are instruments by which *how* to cook dishes can be learned and transmitted amongst people [14], and the internet is an important contemporary medium for their dissemination, such as through recipes websites [15,16]. The internet is accessed daily by 83% of the Brazilian population [17], who spend an average of ten hours connected, while the world average is seven hours a day [18].

Some of the biggest recipe websites on the World Wide Web contain not only recipes posted by the website designers, but also personal recipes uploaded by subscribed users [19]. Research has shown that individuals use the World Wide Web to learn how to cook, to find recipes [20,21,22,23], and to find ideas to plan meals [24,25]. In fact, the digital convenience of being ‘at hand’ has made the internet favored by people when compared to printed sources [22].

The use of the internet by different population groups to obtain information on health, food, and nutrition has increased in recent decades [26,27,28,29]. This medium is actively used to search for information related to food and diet, healthy eating [30], and the relationship between food and health [31]. It has been reported that both the access to recipes and learning about cooking are embedded in sources of information on health, food, and nutrition on the internet [27,32].

If individuals are to obtain knowledge about cooking that enhances health promotion through healthy eating, arguably the content made available must meet guidelines for healthy eating. The concept of healthy eating, however, is not always clear to individuals and is not understood and interpreted in the same way by all [33]. This can lead to different practices in the name of healthy eating [34], as people interpret the concept of healthy eating in complex and diversified ways, reflecting their personal, social, and cultural experiences, as well as the environment in which they live [35].

One of the main challenges in health promotion actions and programs is the development of individuals’ skills to translate dietary information received from a variety of sources into practical information on food selection to achieve healthy eating habits [36]. This is why the World Health Organization has been stressing, since 1992, the importance of understanding the attitudes and beliefs of the population regarding food, nutrition, and health as enablers to the formulation and effective implementation of nutritional guidelines for the promotion of healthy eating [37]. The situation turns out to be even more important in a context where ‘healthiness’ is commonly used as a food-related marketing strategy [38]. The use of nutrient or health claims, such as ‘gluten free’ and ‘lactose free’ on products’ ‘front-of-pack’ labels is rather common in UP foods [39], which can give them a false healthy stereotype [40,41].

As part of the food environment, the internet is increasingly identified as an important influencer of food choices. Health problems related to unbalanced diets can be tackled with initiatives that involve the food environment of individuals, but effective and feasible strategies to facilitate the decision making of individuals must come after identifying the contents conveyed in the food environment, whether physical or digital [42].

Studies on the quality of recipes shared on the internet are scarce (albeit recent) and show that most recipes do not have healthy characteristics when compared to recommendations for healthy eating [19,43], including those that use a ‘healthy’ appeal in their descriptions [19,44,45]. The only two Brazilian studies identified on this topic evaluated recipes from websites and blogs, either aimed at a very specific audience (pre-schoolers) [45], or at social media (YouTube^®^, San Bruno, CA, USA) [43]. Considering the vast sources of culinary recipes online, there is still a gap in the literature which prevents us from knowing whether they are, in fact, contributing to the promotion of healthy eating as recommended by national dietary guidelines.

To our knowledge, this is the first paper that aims to analyze the healthiness of culinary recipes available on the ‘healthy eating’ section of a popular Brazilian recipe-sharing website.

## 2. Materials and Methods

### 2.1. Website Selection

For this cross-sectional study, one website specializing in recipe sharing was purposely selected in October 2019, after a systematic search conducted on the Google^®^ platform. Google^®^ was identified as the most accessed search engine in Brazil, according to Amazon^®^ ranking [46]. Navigating in an anonymous window and using the advanced search option, the term ‘recipes’ (in Portuguese, ‘receitas’) was inserted in the google.com search page. Search filters included language (Portuguese), country (Brazil), and relevance (most relevant). In the literature, we could not find a specific recommendation for how many search results should be ideally assessed for eligibility. A previous study on the same topic as ours analyzed the first 20 results [45], but experimental studies about online searches indicate that in less than 2% of cases, individuals look past the first results page [47]. We conducted a pilot search and observed that after the 50 first links (5 pages), results became irrelevant to our study. Therefore, in this study, without applying a date limit, the first 50 results (links) were accessed by the research team. The eligibility criteria established for the website were: existence of at least one section of recipes named with health-related words and expressions (‘healthy eating’, ‘healthy’, ‘health’, ‘well-being’, or similar) (*n* = 13 excluded); being characterized as collaborative, i.e., user-created content (posted recipes), based on their own personal experiences (*n* = 6 excluded); being solely devoted to sharing recipes and no other type of content (*n* = 3 excluded); not being sponsored by the food industry (*n* = 19 excluded); and not being advertised at the top of the search results (*n* = 8 excluded).

Only one website met the eligibility criteria. TudoGostoso (tudogostoso.com.br) was launched in 2005 and, at the time of the manuscript write-up, claimed to be the largest recipe website in Brazil. The page was created as a collaborative website, a kind of “*shared recipe notebook that brought together traditional family recipes and new discoveries in the kitchen*”. There are more than 196 thousand recipes available, which are accessed by 30 million individuals monthly and by more than 1 million Brazilians daily [48].

### 2.2. Recipes Selection

The third author accessed and downloaded all recipes available on the ‘healthy eating’ section of the website until March 2020 as PDF files. Data collection took place from October 2019 to March 2020. A database in Microsoft Excel 2016 was created to register the following information for each recipe: title, link, ingredients, preparation instructions, and preparation time. Only one recipe was excluded from analysis because it was a homemade cough syrup, i.e., not a culinary recipe. Ingredients were gathered from the recipes’ ingredients list and preparation instructions. The final database was composed of 814 recipes.

### 2.3. Data Analysis

The variables used to characterize the recipes were the recipes’ titles, ingredients, and preparation time. The variables used to assess the recipes’ healthiness were ingredients and preparation instructions.

#### 2.3.1. Recipes’ Characteristics

The recipes’ titles and ingredients were submitted to content analysis for the categorization of the type of culinary preparation (e.g., salad, pudding, etc.) (first author). Words and phrases were coded according to similarity, and then categorized into groups. Weak and general categories were regrouped until strong or terminal categories resulted [49]. Analysis was carried out both deductively and inductively, starting with pre-defined categories: appetizers; breads; cakes and baked goods; homemade ingredients; meat or egg main dishes; non-alcoholic beverages; preserves; puddings; sauces; savory cakes and pies; salads; savory spreads and pâtés; side dishes; snacks and homemade fast foods; soups and creams; and sweet spreads [43].

The recipes’ titles were also content analyzed [49] to identify health-related words and phrases (first and second authors). Expressions such as ‘gluten free’ and ‘lactose free’ were categorized as diet trends because the titles did not mention celiac disease or lactose intolerance as clinical conditions which demand specialized diets. A word cloud was generated to illustrate codes. Bigger font sizes of words or expressions indicated a higher frequency of appearance in the recipes’ titles. Microsoft Office 2016, QDA Miner Lite version 2.0.8., and WordClouds.com were used in the analyses.

#### 2.3.2. Recipes’ Healthiness

The recipes’ ingredients were classified according to the extension and purpose of industrial processing into one of four groups: unprocessed/minimally processed (U/MP); processed culinary ingredient (PCI); processed (P); or ultra-processed foods (UP) [1,3,50] (first and second authors).

U/MP foods included fresh, dry, or frozen fruits or vegetables, grains, legumes, meat, fish, and milk. PCI included table sugar, oils, fats, salt, and other substances extracted from foods or from nature for use in culinary preparations. P foods (manufactured by adding salt, sugar, or other substances to U/MP foods) were canned foods, breads, and cheese. Foods formulated with the addition of substances not usually present in culinary preparations, such as flavoring agents, dyes, sweeteners, emulsifiers, and other additives, were classified as UP [1,3,50].

Ingredients that did not have their preparation described in the recipe (e.g., sweetened condensed milk, mayonnaise sauce) but were available for purchase in industrialized versions were classified as P or UP, according to the predominant characteristics of products available in Brazilian retail outlets. In case of no agreement between the first two researchers who conducted the analysis about the extension and purpose of industrial processing, the ingredient was classified in the group of lower processing [51].

The recipes’ ingredients and preparation instructions were assessed with the Qualitative Framework for the Assessment of Culinary Recipes’ Healthiness [52] (first and second authors). The framework is a validated tool based on national recommendations for healthy eating from both editions of the Dietary Guidelines for the Brazilian Population [1,2]. This tool does not exhaustively evaluate healthy eating recommendations, but it focuses on aspects that are likely to impact the recipes’ overall healthiness. Each recipe was fully read and assessed in a spreadsheet according to the ingredients (presence/absence/types of certain foods and food groups) and cooking methods employed. The spreadsheet was organized according to the framework into nine overarching assessment categories with a total of eleven components: whole cereals, breads, and pasta; fruits; vegetables; legumes; nuts and seeds; meats and eggs; added fat; sauces; seasonings; foods with high sugar concentration; and cooking method. Each component was evaluated as either positive or negative, i.e., recommended or not recommended for healthy recipes. A negative criterion was not given with the aim of condemning a certain characteristic of the recipe, but rather to raise awareness about critical aspects regarding the promotion of healthier eating habits. The categories of fruits, vegetables, and legumes; nuts and seeds; and foods with high sugar concentration were assessed in every type of recipe, and the remaining categories were assessed whenever they were present in the recipe [52].

### 2.4. Data Treatment

Ingredients used twice in the same recipe were counted as one (e.g., sugar used in a cakes’ batter and icing). Ingredients not mentioned in the ingredients’ list but cited in the preparation instructions section were included in the analysis. When more than one option of ingredient was mentioned, only the first was considered for analysis (e.g., butter or margarine = butter). Ingredients cited as ‘optional’ were not assessed. Alcoholic beverages mentioned as ingredients of recipes were excluded from the analysis.

Two researchers experienced in the adopted methods were involved in all steps of the analyses. For each analysis, 10% of the recipes from the dataset were randomly selected (randomizer.org (accessed on 18 August 2021)) to be independently checked for agreement (first and second authors). The weighted kappa test of agreement between researchers on the assessment of the ingredients’ extension and purpose of industrial processing was 0.96. On the application of the Qualitative Framework for the Assessment of Culinary Recipes’ Healthiness, agreement ranged between 0.90 and 1.0 (kappa and weighted kappa) between researchers, indicating an almost perfect agreement [53]. Divergences were firstly discussed between the first two authors and in case of no agreement, they were resolved with the participation of the last author.

### 2.5. Statistical Analysis

Qualitative dichotomous and polytomous variables are presented in absolute and relative frequencies. Quantitative variables are presented as median and interquartile range (IQR), as they were not normally distributed when assessed by the Shapiro–Wilk test (considering an alpha of 0.05 as the significance level). Stata SE version 13.0 (College Station, TX, USA) was used for the analysis.

## 3. Results

### 3.1. Recipes’ Characteristics

The recipes’ preparation time ranged from 1 to 180 min (IQR = 10; 40, *n* = 814). The most frequent category of recipes was ‘cakes and baked goods’ (Table 1).

**Figure 1:**
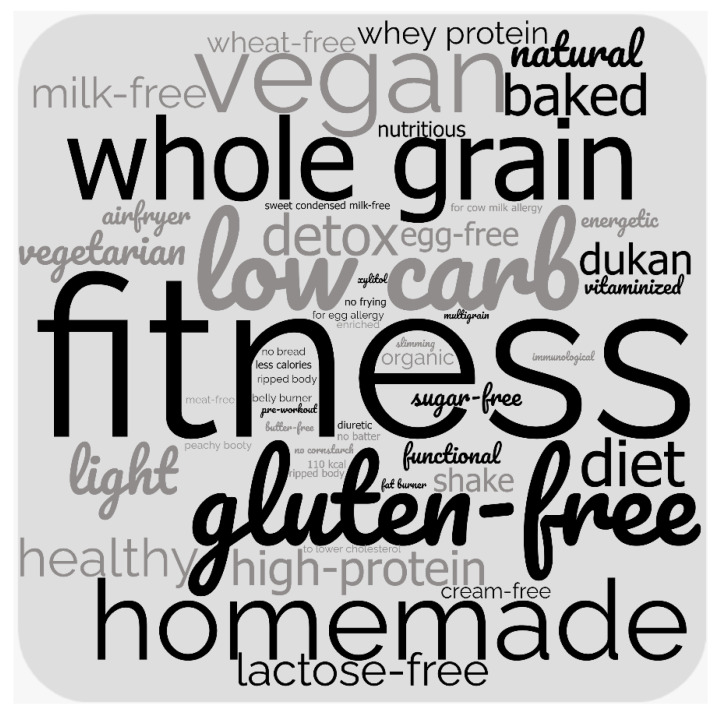
Word cloud generated with codes from content analysis of the titles from 814 culinary recipes available on the ‘healthy eating’ section of a popular Brazilian recipe-sharing website, October 2019–March 2020. The bigger the font-size of each word or expression, the more frequently it appeared in recipes’ titles.

Regarding the recipes’ titles, 564 terms referring to health were coded (Figure 1) and organized into nine categories: diet trends (*n* = 155 codes, e.g., gluten-free, dukan, low-carb, detox); appeal to body image and physical exercise (*n* = 136 codes, e.g., fitness, ripped body, belly burner); appeal to the absence or reduction in ingredients or nutrients (*n* = 69 codes, e.g., diet, sugar-free); production practices and cooking methods (*n* = 54 codes, e.g., organic, not fried); vegetarian and vegan (*n* = 51 codes); whole grain (*n* = 42 codes); health and nutrition (*n* = 32 codes, e.g., vitaminized, nutritious); appeal to the presence or increase in ingredients or nutrients (*n* = 25, e.g., high protein, whey protein); and natural (*n* = 4 codes).

### 3.2. Recipes’ Healthiness

From the total 5887 ingredients analyzed, the majority were U/MP (62.4%, *n* = 3673) and PCI (23.2%, *n* = 1364). Ingredients classified as P (6.1%, *n* = 357) and UP (8.4%, *n* = 493) were less frequent. The ten most frequent U/MP ingredients in the sample were, in decreasing order of appearance: egg, onion, water, oat, garlic, banana, pepper, tomato, carrot, and cinnamon. The ten most frequent UP ingredients in the sample were, in decreasing order of appearance: artificial sweetener, UHT cream, margarine, bottled coconut milk, cheese spread, whey protein, industrialized tomato sauce, textured soy protein, shoyu sauce, and vanilla essence.

Positive aspects regarding the recipes’ healthiness include a high frequency of whole cereals, breads and/or pasta (72.0%, *n* = 280), and the use of either lean cuts of meat or eggs (86.6%, *n* = 129). The exclusive use of industrialized seasonings (3.1%, *n* = 11) and of frying as a cooking method (3.1%, *n* = 22), as well as the presence of foods with a high sugar concentration (23.6%, *n* = 192), were not frequent. Negative aspects to be highlighted are the low presence of fruits (32.7%, *n* = 263), legumes (4.7%, *n* = 380), nuts and seeds (18.4%, *n* = 150), and the presence of margarine (8.3%, *n* = 34) in the recipes. The categories that presented the most evenly distributed positive and negative criteria were the presence of vegetables and different types of sauces (Table 2).

## 4. Discussion

This study analyzed the healthiness of recipes available on the ‘healthy eating’ section of a popular Brazilian recipe-sharing website, using national dietary guidelines as references.

UP foods represented 8.4% of all ingredients in the ‘healthy’ recipes. As the Brazilian Guidelines for Healthy Eating determine that UP food consumption should be altogether avoided [1], people looking for healthy recipes on the internet might end up being misled in their effort to follow the national guidelines. A high consumption of UP foods has been associated with chronic non-communicable diseases and all-cause mortality [54,55], but it constitutes a substantial portion of Brazilians’ diets (18.4% of daily calories) [56]. Research has shown that, although some people notice the presence of some food additives and hydrogenated fats as indicators of UP food unhealthiness, they do not spend much time reading ingredients lists, and tend to rely on simple cues when choosing, such as package design and brand [40]. In fact, some UP foods are stereotypically perceived as healthy, being seen as foods that do not lead to weight gain or have a small weight-loss effect [41].

Artificial sweeteners were one of the most frequent UP ingredients in the sample, as several recipes used them to replace sugar (a PCI) in the main recipe category shared (cakes and baked goods). This explains the low presence of foods with a high sugar concentration observed in the analysis with the framework, which, in theory, should be a positive result. Artificial sweeteners were developed by the food industry in the context of a dramatic increase in the incidence of obesity to reduce calorie and, specifically, added sugars intake [57,58]. However, evidence on the negative health effects of artificial sweeteners’ intake is accumulating, such as the association with cardiovascular disease and mortality [59], and with increased cancer risk [60]. Unfortunately, many in the general public still adopt the use of sweeteners as a short-term strategy to reduce dietary sugar and for weight management [61].

The framework analysis identified the presence of margarine in some recipes. It was also one of the most frequent UP ingredients in the sample. Margarine was developed by the food industry in the 19th century as a cheap substitute for butter, but it was later marketed as an option to lower saturated fat intake because of its vegetable origin [57]. As an UP food mainly composed of hydrogenated vegetable fat or interesterified oils [1], it should not be used in culinary preparations if the national guidelines are to be followed. Trans fatty acids have many negative impacts on health [62] and are related to all-cause mortality, and cardiovascular disease [63].

The common use of artificial sweeteners and margarine by users of the investigated website may indicate that reductionist conceptions—‘butter and sugar are unhealthy’—are still leading to the substitution of processed culinary ingredients by UP foods. If used in moderation and in recipes based on U/MP foods, PCI can indeed be part of a healthy diet [1,3,64]. Awareness of the concept of healthy eating, especially as proposed by the latest edition of the Brazilian dietary guidelines, which is based on food processing, may still find some barriers. Even health professionals struggle to correctly classify foods according to their processing characteristics [65]. Adding to this is individuals’ inability to judge the veracity of marketing strategies employed by the food industry [31].

Many recipes’ titles alluded to health by using words or expressions from diet trends (gluten-free, dukan, low-carb, detox), or appealing to body image and physical exercise (fitness, belly burner). Whey protein was one of the most frequent UP foods present in the sample. This suggests that the individuals posting recipes on the website may associate healthiness to weight loss and physical appearance, which does not resonate with the perspective of national dietary guidelines [1]. Beliefs and attitudes towards healthy eating are linked to psychological self-perception and personal ways of thinking. For some, the belief of improving one’s physical appearance and attractiveness facilitates healthy eating [66].

It is in this scenario that restrictive diet trends, which have no proven long-term benefits or sustainability [67,68,69], continue to emerge, grow, and sustain themselves. Like in a vicious cycle, the expectation of rapid weight loss, and body and beauty perceptions of society are reasons that individuals give for being on a popular diet [70]. The growing offer of products from health, wellness, and sports food sectors [39,67,71] may be, in turn, also feeding individuals’ flawed perception of healthy eating. Whey protein, for example, is on the rise as a food component following fitness trends because of the association of high-protein foods with satiety and lean body mass gain or maintenance [67]. The Brazilian dietary guidelines alert that there is much information about food, diet, nutrition, and health on all sorts of media; however, most of their usefulness is questionable as they confuse healthy eating with weight-loss dieting regimes, and some informative notices are actually a veiled form of advertising UP foods [1].

Lastly, although U/MP ingredients were highly frequent in the sample analyzed, the application of the framework evidenced that fruits, legumes, nuts, and seeds were rather absent in the recipes. Therefore, some key food groups for healthy eating are not being as valued as other aspects, such as favoring whole cereals and lean cuts of meat. This result mirrors the results of epidemiological studies of Brazilians’ diets, which is reportedly low in fruits, legumes, nuts, and seeds [72], and may indicate individuals’ difficulty to incorporate those foods in culinary recipes, even though they are frequently associated with healthiness [73]. Individual, social, and environmental barriers besides knowledge about healthy eating [74] may be behind the low frequency of those U/MP foods in so-called ‘healthy’ recipes. One of them refers to the formation of eating habits during childhood through a lack of exposure to foods, such as fruits, vegetables, legumes, nuts, and seeds. Another barrier frequently linked to the ingestion of U/MP foods is the need for preplanning, which is further linked to time constraints and exacerbated by an overall lack of cooking skills [66]. A high consumption of fruits, vegetables, and legumes is an integral part of healthy eating patterns [75] and a basic dietary priority to reduce chronic disease risk [76]. A higher consumption of fruits, vegetables and legumes is associated with the prevention of chronic diseases, such as cardiovascular diseases and the mortality rates related to them [77,78]. The ingestion of nuts is also associated with a reduced risk of cardiovascular disease and diabetes [79,80].

Comparisons with previous research on the healthiness of recipes shared on websites devoted to this are complex, because this is a relatively new field of research with only a few studies published. Additionally, such studies addressed nutrient content to assess recipes’ healthiness [19], while our study did a qualitative-based assessment. Studies on the subject also focused on different data sources, such as ‘clean eating’ blogs [44] or recipes for pre-schoolers [45]. Nevertheless, our study agrees with previous research, where most recipes online were found to be unhealthy according to the World Health Organization and the United Kingdom Food Standards Agency’s recommendations [19], including those that use a ‘healthy’ appeal in their descriptions [19,44,45]. Previous research in the Brazilian context, conducted on YouTube^®^ and not focused on ‘healthy recipes’, also identified the presence of UP foods as ingredients and a scarcity of fruits, legumes, nuts, and seeds [43]. Our results help to fill a gap in the literature, revealing not only that the recipes shared as ‘healthy’ deviate from dietary guidelines, but also appeal to diet trends, body image, and physical exercise.

In a nutshell, interventions and health education activities are needed to tackle specific points when encouraging individuals to cook and use the internet as a source of information. Firstly, people need to be alerted that even recipes branded as ‘healthy’ are not necessarily following dietary guidelines for healthy eating. Secondly, it is important to promote cooking in a healthy way, without the addition of UP foods as ingredients to (artificially) enhance flavor or as shortcuts to shorten preparation time. It is also relevant to demonstrate how it is possible to incorporate more fruits, legumes, nuts, and seeds in recipes to improve their quality. As stated earlier, a negative criterion was given with the aim of raising awareness about the critical characteristics of recipes, and therefore promote healthier eating habits. Each negative criterion is considered a point of possible improvement through the substitution, addition, or exclusion of ingredients, and/or the alteration of the cooking method. We highlight that to make an original recipe healthier, not all the negatively assessed components should be changed. Finally, as proposed by Marsola et al., it is necessary not only to disseminate information about the composition, degree of processing, and origin of foods, but to take into consideration beliefs and values individuals are giving to healthy eating [41]. In the future, partnerships between website developers and researchers can be established to develop algorithms based on national dietary guidelines to guide users towards healthy recipes.

One of the study’s limitations is the adoption of a conservative criterion to classify ingredients by the extension and purpose of industrial processing. This criterion was used because ingredients were identified from recipes, and not from information on labels. Although minimally employed, this strategy may have led to an underestimation of the number of UP ingredients [51]. Data were retrieved from only one website, which compromises extrapolation to other websites and contexts. Nevertheless, the website was claimed to be the largest in Brazil, and the sample size was robust, quality control was rigorous, and a validated framework was employed in the analyses. Finally, recipes were analyzed as a whole; therefore, we were not able to provide an assessment of each recipe category’s healthiness.

## 5. Conclusions

The recipes’ titles appealed to diet trends, body image, and physical exercise. Recipes employed UP foods as ingredients, but the use of fruits, legumes, nuts, and seeds was not encouraged. Therefore, the ‘healthy’ recipes available on the Brazilian recipe-sharing website presented many aspects not in accordance with national dietary guidelines. Findings are relevant to inform health professionals and policymakers to act towards educating individuals on how to choose proper healthy recipes to effectively promote cooking as a healthy eating strategy.

## Figures and Tables

**Table 1:** Categories of 814 culinary recipes available on the ‘healthy eating’ section of a popular Brazilian recipe-sharing website, October 2019–March 2020.

Recipes’ Categories	%	*n*
Cakes and baked goods	22.4	182
Side dishes	11.2	91
Snacks and homemade fast foods	10.4	85
Meat or egg main dishes	9.8	80
Puddings	9.0	73
Breads	6.5	53
Non-alcoholic beverages	5.4	44
Vegetarian and vegan dishes	5.2	42
Savory cakes and pies	4.9	40
Salads	4.4	36
Homemade ingredients	4.2	34
Appetizers	2.0	16
Savory spreads and pâtés	1.7	14
Sauces	1.1	9
Sweet spreads	0.7	6
Soups and creams	0.7	6
Preserves	0.4	3
Total	100	814

**Table 2:** Healthiness of 814 culinary recipes available on the ‘healthy eating’ section of a popular Brazilian recipe-sharing website according to the Qualitative Framework for the Assessment of Culinary Recipes’ Healthiness, October 2019–March 2020.

Category	Description of Components	Criteria	%	*n*
Foods with high starch content (*n* = 389)	Exclusive presence of whole cereals, breads, and/or pasta	+	57.6	224
	Mixed presence of whole and refined cereals, breads, and/or pasta	+	14.4	56
	Exclusive presence of refined cereals, breads, and/or pasta	−	28.0	109
Fruits, vegetables, and legumes	Presence of vegetables	+	42.9	349
	Absence of vegetables	−	57.1	465
	Presence of legumes	+	4.7	38
	Absence of legumes	−	95.3	776
	Presence of fresh, frozen, or dried fruits	+	32.7	263
	Absence of fresh, frozen, or dried fruits	−	67.3	542
Nuts and seeds	Presence of nuts and seeds	+	18.4	150
	Absence of nuts and seeds	−	81.6	664
Meats and eggs (*n* = 149)	Exclusive presence of lean cuts of meat, poultry cuts without skin, fish, seafood, and/or eggs	+	75.2	112
	Mixed presence of lean cuts of meat, poultry cuts without skin, fish, seafood and/or eggs and non-lean cuts of meat, poultry cuts with skin and/or processed meats	+	11.4	17
	Exclusive presence of non-lean cuts of meat, poultry cuts with skin, and/or processed meats	−	13.4	20
Fats (*n* = 408)	Exclusive use of vegetable oils, butter and/or lard in place of margarine	+	91.7	374
	Presence of margarine	−	8.3	34
Sauces (*n* = 78)	Exclusive presence of tomato sauce with herbs	+	48.7	38
	Mixed presence of tomato sauce with herbs and white sauce, with mayonnaise or cheese	+	7.7	6
	Exclusive presence of white sauce, with mayonnaise or cheese	−	43.6	34
Seasonings (*n* = 349)	Exclusive presence of olive oil, lemon, and/or fresh or dried herbs	+	74.0	258
	Mixed presence of olive oil, lemon, and/or fresh or dried herbs, and industrialized spices, sauces, and/or broths	+	22.9	80
	Exclusive presence of industrialized spices, sauces and/or broths	−	3.1	11
Sugars	Presence of foods with high sugar concentration	−	23.6	192
	Absence of foods with high sugar concentration	+	76.4	621
Cooking method (*n* = 706)	Use of steam, cooking in water without or with little fat, stewing, roasting, broiling, sautéing	+	96.9	684
	Use of steam, cooking in water without or with little fat, stewing, roasting, broiling, sautéing?and/or frying	−	3.1	22

Fruits, vegetables, and legumes; nuts and seeds; and sugars categories are mandatorily assessed in all recipes. The remaining categories are assessed only when applicable. Criteria: + and − indicate recommended and not recommended components for healthy recipes, respectively [52].

## Data Availability

Not applicable.
